# Anti‐Inflammatory Activity and Mechanism of Stearic Acid Extract From Purslane

**DOI:** 10.1002/fsn3.70596

**Published:** 2025-07-10

**Authors:** Xiao‐Min Lin, Ai‐Jing Chen, Hui‐Min Zhang, Zhi‐Zhuo Kuang, Rui Chen, Meng‐Die Wu, Jie Chen, Xin Ma, Lei Zhao, Yan Xing, He Ni

**Affiliations:** ^1^ Guangdong Provincial Key Lab of Biotechnology for Plant Development, School of Life Sciences South China Normal University Guangzhou China; ^2^ College of Food Science South China Agricultural University Guangzhou China; ^3^ Guozhen Health Technology (Beijing) Co. Ltd Beijing China; ^4^ School of Food and Biology Guangdong Polytechnic of Science and Trade Guangzhou China

**Keywords:** anti‐inflammatory activity, inflammatory bowel diseases, purslane, stearic acid

## Abstract

Purslane, as a traditional edible and medicinal herb, has been proven to have good anti‐inflammatory activity. In this study, it was clarified that stearic acid was the main anti‐inflammatory substance in purslane and could decrease the secretion of inflammatory cytokines, tumor necrosis factor‐α (TNF‐α), interleukin‐6 (IL‐6), and interleukin‐10 (IL‐10) in lipopolysaccharide (LPS)‐stimulated RAW 264.7 cells. Meanwhile, the inhibitory effect of liposoluble extract of purslane (PLE) and stearic acid on LPS‐induced ulcerative enteritis was determined. LPS‐induced enteritis, including body weight loss, reduced weight of the small intestine, and histological intestinal damage, was significantly ameliorated in mice fed PLE and stearic acid. In particular, PLE could significantly reduce the levels of TNF‐α, IL‐6, and interleukin‐1β (IL‐1β) in the small intestine through NF‐κB and MAPK signaling pathways. Thus, our results proved that purslane could be considered a natural anti‐inflammatory medicine to combat LPS‐induced inflammation and provide a theoretical basis for future applications in the field of medicine or functional food.

## Graphical abstract

- PSA was a safe and effective anti‐inflammatory extract.
- PLE treatment alleviated LPS‐induced enteritis symptoms in mice.
- PLE significantly reduced the levels of TNF‐α, IL‐6, and IL‐1β in the small intestine through the NF‐κB and MAPK signaling pathways.

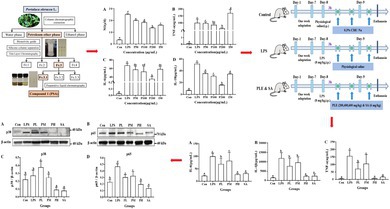

## 1 Introduction

Inflammation serves as a fundamental biological response to harmful stimuli, such as pathogens, damaged cells, or irritants, playing a critical role in the body's immune defense (Badraoui et al. 2021). However, when inflammation becomes chronic, it can contribute to the pathogenesis of numerous diseases, including cardiovascular diseases, diabetes, cancer, and autoimmune disorders. Inflammatory bowel disease (IBD) is an idiopathic disease associated with chronic inflammation of the intestine, including Crohn's disease (CD) and ulcerative colitis (UC) (Grossberg et al. 2022). Its pathogenesis remains largely unknown and may provoke dysregulation of the intestinal immune environment, cause intestinal mucosal damage, and induce the inflammatory response while releasing pro‐inflammatory chemokines, which are manifested by inflammatory cell infiltration, intestinal villus degeneration, necrosis, and shedding (Del Sordo et al. 2022; El Menyiy et al. 2022).

Currently, approved biological and small‐molecule therapies for IBD are anti‐TNF drugs, anti‐integrin therapies, anti‐IL‐12/23 therapy, JAK inhibitors, and S1P modulators (Grossberg et al. 2022). However, these treatments have drawbacks like low efficacy and poor safety. Prolonged or high‐dose drug treatment may cause accumulation and significant side effects, including increased risk of cardiovascular and cerebrovascular problems, and potential cancer development. Hence, novel candidates with high efficacy and minimal side effects are urgently needed for the medical treatment of IBD. For thousands of years, natural ingredients and herbs have been used to heal and prevent diseases in humans (Cheung 2011; Rahmouni et al. 2017). Plant bioactive ingredients with intestinal anti‐inflammatory activity have been developed for IBD treatment, including flavonoids, terpenes, and fatty acids. There is no doubt that natural anti‐inflammatory substances are believed to be safer and more effective than synthetic drugs, offering an opportunity to find natural anti‐inflammatory agents from traditional herbal plants.

Purslane ( *Portulaca oleracea* L.), a traditional edible and medicinal herb, is commonly distributed in tropical and subtropical regions. It is widely consumed as food in the food industry (Srivastava et al. 2023; Zhou et al. 2015). In traditional Chinese medicine, purslane is recognized as a natural antibiotic due to its ability to clear heat, detoxify, lower blood pressure, and stop diarrhea. Numerous studies have shown that bioactive compounds extracted from purslane possess anti‐inflammatory, pain‐relieving, liver‐protective, antifungal, and antioxidant properties (Lu et al. 2022). Our preliminary research confirmed that the liposoluble extract of purslane (PLE) had good anti‐inflammatory activity, among which the main active component was stearic acid (Zhang et al. 2024). Based on this foundation, this study explored the anti‐inflammatory activity of stearic acid and the mechanism of its extract from purslane on protective effect against IBD. This research lays the foundation for the application of stearic acid and purslane extract in the treatment of inflammation, while also providing a theoretical basis for enhancing the development value of purslane.

## 2 Material and Methods

### 2.1 Chemicals and Reagents

Stearic acid (SA, purity > 98%) and lipopolysaccharides (LPS) (from *Escherichia coli* strain 055: B5) were provided by Sigma Aldrich Co. (Santa Clara, CA, USA). Cell counting kit‐8 (CCK‐8) was purchased from Boster Biological Technology Co. Ltd. (CA, USA). Dulbecco's modified Eagle's medium (DMEM), streptomycin sulfate, fetal bovine serum (FBS), and penicillin were obtained from Thermo‐Fisher Scientific. Enzyme‐linked immunosorbent assay (ELISA) test kits such as TNF‐α, IL‐1β, IL‐6, and IL‐10 were provided by Neobioscience Technology Co. (Shenzhen, China). All the other chemicals used were of analytical grade.

### 2.2 Preparation of Stearic Acid Extract From Purslane

Stearic acid extract from purslane (PSA) containing over 50% SA was prepared as previously reported by Zhang et al. (2024) with minor modifications (Zhang et al. 2024). The appropriate amount of sample was accurately weighed, then extracted with a petroleum ether–ethanol (8:2) solution with 3 times the volume of the material for 2 h at room temperature using column chromatographic extraction, which was PLE containing 10% SA (Chen et al. 2019). Then PLE was separated by silica gel column chromatography twice using gradient elution of petroleum ether and ethyl acetate (18:3, 9:3), followed by isocratic elution of dichloromethane: methanol (100:1). Fraction 3.1 was collected as PSA.

### 2.3 Cell Culture

Murine macrophage cell line RAW264.7 cells were purchased from the Cell Bank of the Chinese Academy of Sciences and cultured in DMEM medium with 10% fetal bovine serum, 100 U/mL penicillin, and 100 μg/mL Streptomycin sulfate in a humidified atmosphere of 5% CO_2_ at 37°C and subcultured every 3 days.

### 2.4 Cell Viability and Nitric Oxide (NO) Production

The RAW264.7 cell experiment was performed following the method of Xie et al. (Xie et al. 2023). After incubation with 50, 100, and 200 μg/mL of PSA and 50 μg/mL of indomethacin for 24 h, cell viability and NO content in the supernatant were evaluated using the CCK‐8 and Griess Assay Kit (Beyotime, Shanghai, China).

### 2.5 Enzyme‐Linked Immunosorbent Assay

The levels of cytokines TNF‐α, IL‐6, IL‐1β, and IL‐10 in RAW264.7 cells and the intestinal tissues of Balb/c mice were measured by using enzyme‐linked immunosorbent assay (ELISA) kits (Neobioscience, Shenzhen, China) following the instructions provided by the manufacturer.

### 2.6 Animals and Experimental Design

The experiment was performed according to the method of Wang et al. with minor modifications (Wang et al. 2022). A total of 36 male Balb/c black mice (aged 5–6 weeks) were procured from Guangdong Medical Laboratory Animal Center (Guangdong, China), with Animal License No. SCXK (Yue) 2022‐0002, and fed at South China Agricultural University (Guangzhou, GD, China, Animal License No. SYXK (Yue) 2022–0136). All experiments were conducted in compliance with the guidelines of the Experimental Animal Center Welfare Committee of the South China Agricultural University.

**FIGURE 1:**
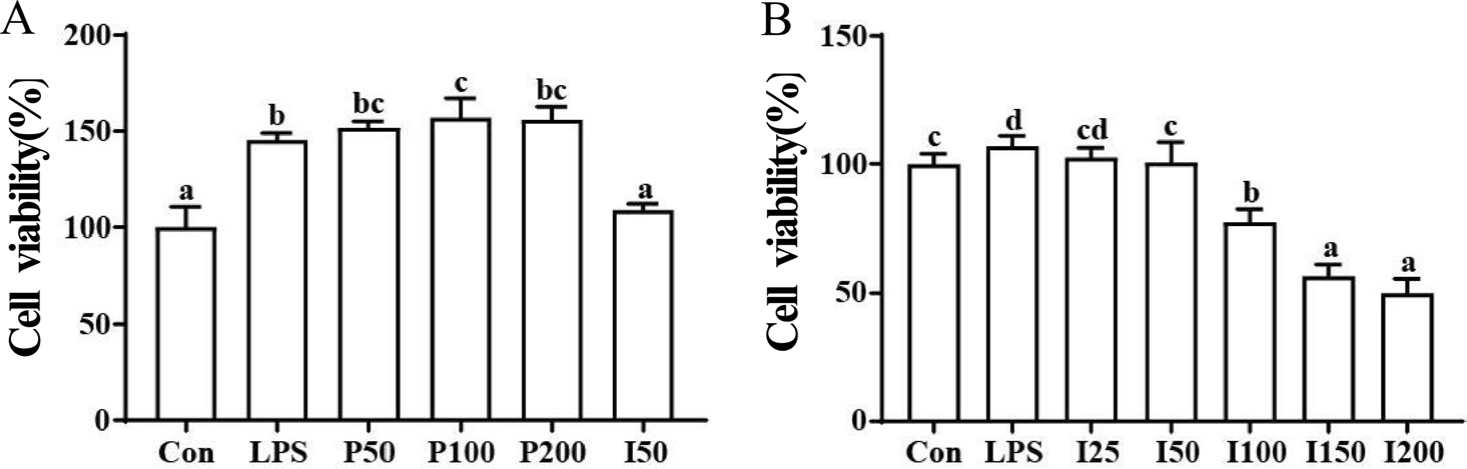
The cytotoxicity of different concentrations of PSA (A) and indomethacin (B) to RAW264.7 macrophages.

**FIGURE 2:**
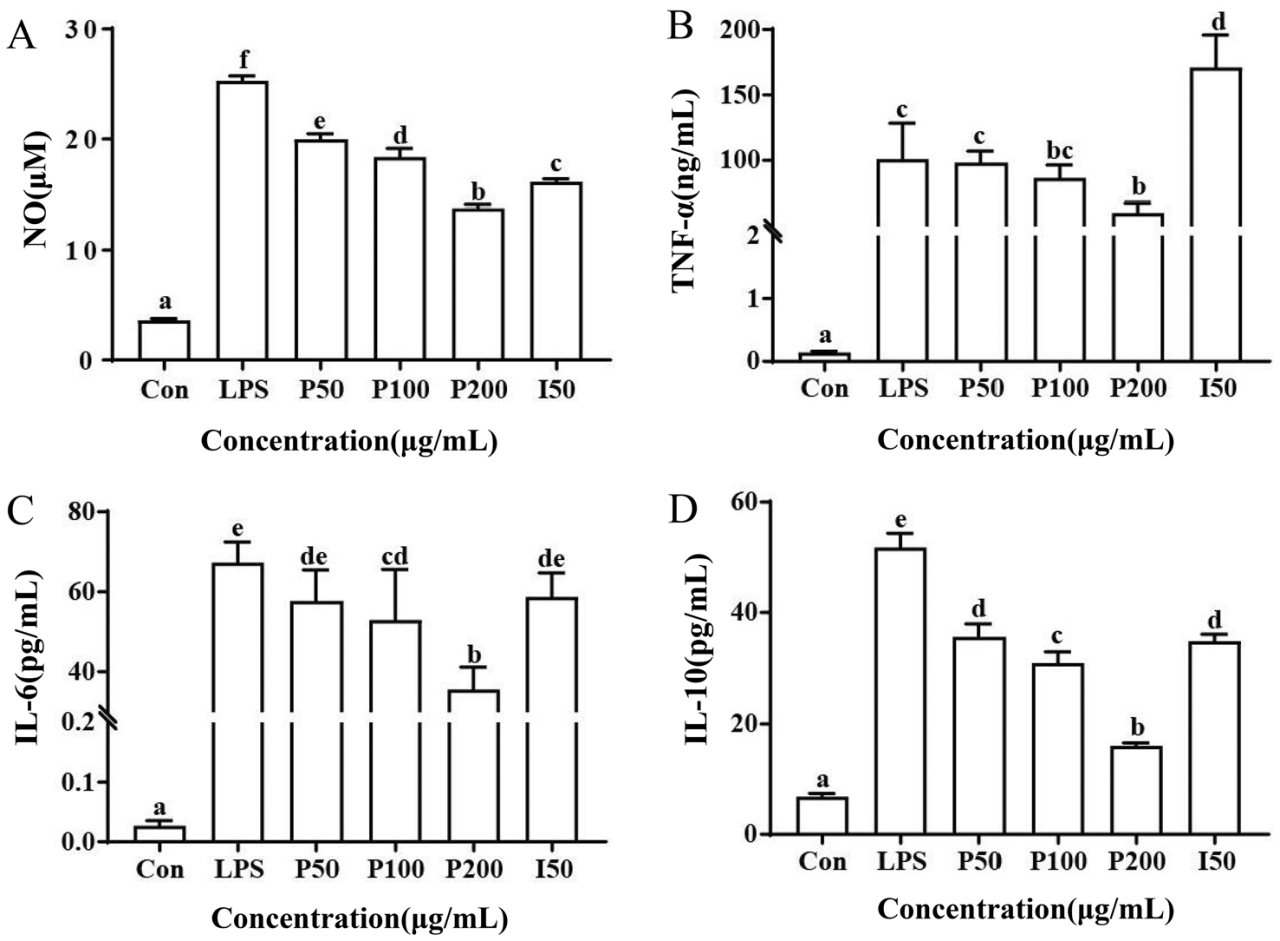
Effect of PSA on the release of NO (A), TNF‐α (B), IL‐6 (C), and IL‐10 (D) in RAW264.7 macrophages.

**FIGURE 3:**
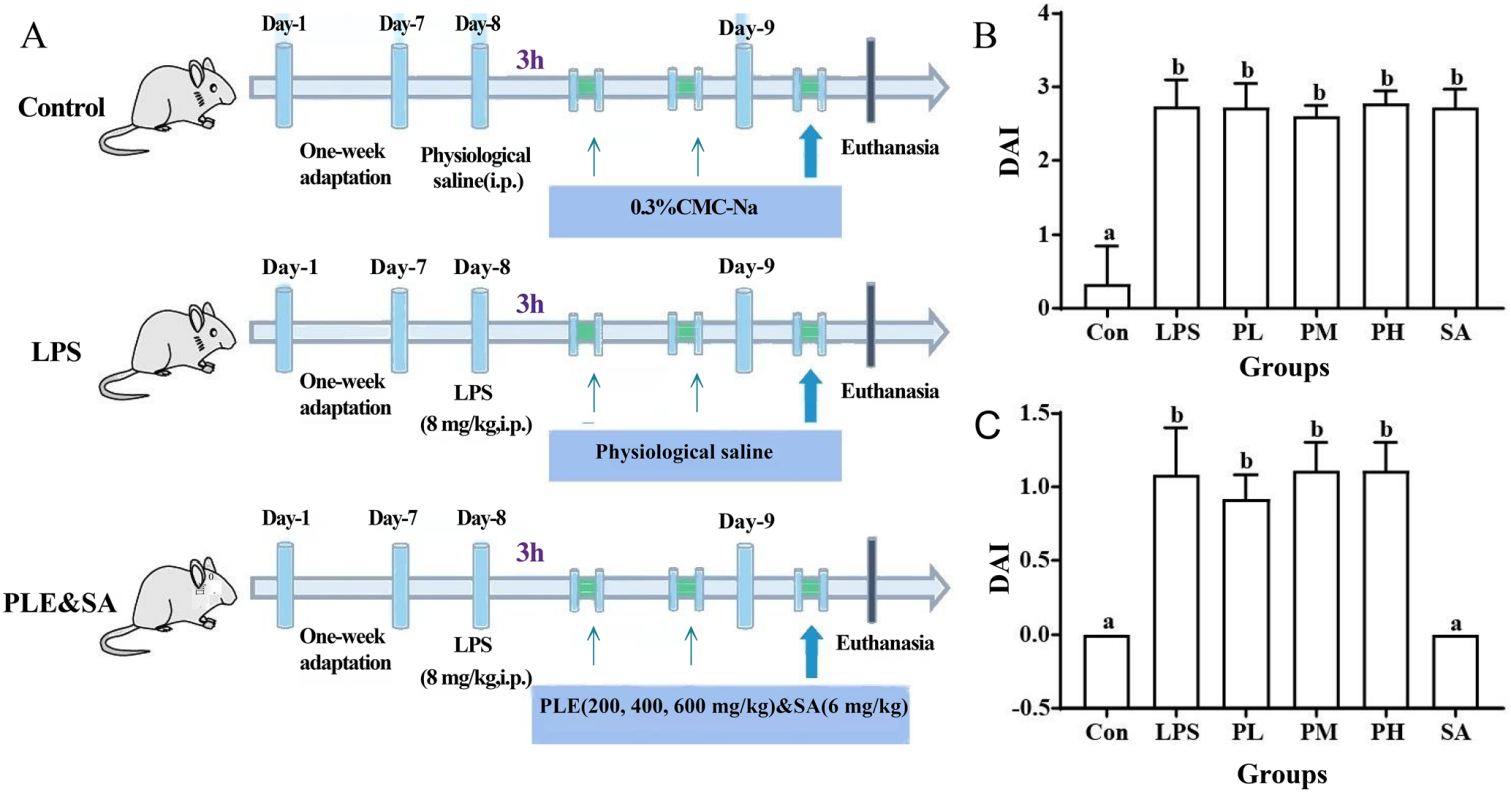
Effects of PLE and SA on LPS‐induced small intestine injuries in mice. (A) Diagram of the animal experimental scheme; DAI score before (B) and after (C) administration of PLE and SA.

The mice were kept in a temperature‐controlled environment of 22°C ~ 24°C with a 12‐h light‐controlled cycle at 25°C and ad libitum food and water. After a week of adaptation, the mice were randomly divided into six groups (*n* = 6). The groups were designated as the control group (Con), the model group (LPS), the low, medium, and high doses of PLE group (PL, PM, and PH), and the standard group (SA), respectively. As shown in Figure 3A, mice in all groups except the Con group were injected intraperitoneally with 8 mg/kg LPS. 3 h later, the Con group was given 0.3% CMC‐Na by gavage, and the LPS group was gavaged with physiological saline as a vehicle. The other groups were treated with 200, 400, and 600 mg/kg PLE and 6 mg/kg SA twice daily by gavage. After 24 h, mice were stunned with carbon dioxide and executed. The entire small intestine of each mouse was dissected, and its length and weight were measured. Tissues were collected for pathological studies. During this period, the body weight (BW) and disease activity index (DAI) of mice were monitored. The overall DAI was calculated by averaging the scores of weight loss, stool consistency, and gross bleeding for each mouse in each group.

### 2.7 Histologic Analysis

The Intestinal tissues were fixed in 4% paraformaldehyde, then dehydrated and embedded in paraffin. Sections (4 μm thick) were stained with H&E (Hu et al. 2021). The tissue architectures were examined using a light microscope (Nikon Eclipse E100, Nikon, Japan).

### 2.8 Western Blot Analysis

Small intestine tissue was taken and ground with PBS buffer (Biosharp, China) to prepare a total intestinal protein extract. Then the protein samples were quantified using the BCA method (Shanghai Yuanye Bio‐Technology, China). Subsequently, the protein samples were subjected to electrophoresis on a 10% sodium dodecyl sulfate‐polyacrylamide gel electrophoresis (SDS‐PAGE) and then transferred to polyvinylidene difluoride (PVDF) membranes (Millipore, USA). Then the membrane was blocked overnight with 5% skim milk powder at 4°C. The next day, after washing the membrane with tris‐buffered saline (TBS), the primary antibody (p38 and p65, 1:1000) was tagged at room temperature for 2 h, followed by the secondary antibody (1:5000) at room temperature for 1 h with gentle shaking. Protein bands were detected using enhanced chemiluminescent reagent. Image J software was used to quantify the integrated density of each band. β‐actin was set as the internal control.

### 2.9 Statistical Analysis

The results are shown as mean ± SD for all of the representative experiments. Multiple comparisons between the experimental groups were distinguished by one‐way ANOVA. The level of significance was set at *p* < 0.05. For statistical analysis, SPSS 26 and Origin 2018 were used.

## 3 Results and Discussion

### 3.1 Effects of PSA on LPS‐Induced NO and Inflammatory Cytokines Production in RAW264.7 Macrophages

It was confirmed that PLE possessed good anti‐inflammatory activity in our preliminary research (Zhang et al. 2024). To further explore the anti‐inflammatory activity and mechanism of PSA, different concentrations of PSA were added to LPS‐induced RAW264.7 macrophages. As a kind of natural plant extract, PSA did not exert cytotoxicity to RAW264.7 macrophages within the range of action concentrations (< 200 μg/mL), which showed higher safety and biocompatibility than the common anti‐inflammatory drug Indomethacin (Figure 1). Then the effects of PSA on LPS‐induced NO and inflammatory cytokine production in RAW264.7 macrophages were assayed. As shown in Figure 2A, when cells were stimulated with LPS, the level of NO was increased to 23.63 ± 0.6 μM in the culture medium. After being added for 24 h, PSA could significantly inhibit NO production, and PSA inhibited NO release in a dose‐dependent manner. Furthermore, the inhibition effect of 200 μg/mL PSA was higher than indomethacin, which exerted good anti‐inflammatory activity in the safe dosing concentrations.

Cytokines, serving as pivotal effector and messenger molecules within the immune system, play a critical role in orchestrating the body's immune response and maintaining tissue homeostasis. Their dual nature underscores their importance in both the protection against pathogens and the pathogenesis of various diseases (Ouyang and O'Garra 2019). The release of cytokines is an important step in regulating host immune responses to inflammation (Shou et al. 2019). To assess the anti‐inflammatory effect of PSA in LPS‐stimulated RAW264.7 cells, the release of pro‐inflammatory cytokines IL‐6 and TNF‐α, as well as the anti‐inflammatory cytokine IL‐10, was measured by ELISA kits. As expected, LPS significantly increased the levels of IL‐6, IL‐10, and TNF‐α in RAW264.7 cells. After PSA treatment, the production of cytokines was significantly inhibited in a concentration‐dependent manner (Figure 2B,C,D). At the highest concentration of PSA (200 μg/mL), the levels of TNF‐α, IL‐6, and IL‐10 were diminished by approximately 45%, 50%, and 70%, respectively. PSA had similar or higher inhibitory effects on TNF‐α, IL‐6, and IL‐10 expression than the indomethacin group at the same concentration (50 mg/mL). Furthermore, PSA exerted higher inhibitory effects without cytotoxicity at higher concentrations compared with indomethacin. TNF and IL‐6, as pro‐inflammatory cytokines produced by monocytes and M1 macrophages, may contribute to the development of non‐resolving inflammation and play important roles in the pathophysiology of various non‐communicable inflammation‐related diseases (Netea et al. 2017). It was researched that the secretion of TNF and IL‐6 by monocytes is substantially influenced by certain fatty acids (Hung et al. 2023). Unlike other saturated fatty acids (such as palmitic acid), SA has some beneficial effects on human health through our diet, especially lowering blood pressure, improving heart function, and reducing the risk of cancer (Nůsková et al. 2021). SA has been shown to decrease the production of inflammatory cytokines and exert beneficial effects on inflammation‐related diseases (Pan et al. 2010). Meanwhile, IL‐10, as an anti‐inflammatory cytokine, has a two‐way immunoregulatory effect, and the different expression patterns of IL‐10 receptors lead to different immune activities of IL‐10 in different microenvironments, cells, and pathological conditions. The research by Murray et al. suggested that the anti‐inflammatory effects of IL‐10 on macrophages were primarily due to its influence on TNF‐α gene transcription (Murray 2005). Therefore, the reduction in IL‐6, IL‐10, and TNF‐α induced by PSA could have anti‐inflammatory effects in vitro.

### 3.2 PLE Treatment Alleviated LPS‐Induced Enteritis Symptoms in Mice

Purslane, as a “power food of the future,” its nutritional and medicinal properties have always been underestimated in the food and other related industries (Gonnella et al. 2010). It has been demonstrated that PLE and PSA could inhibit the release of NO and the secretion of inflammatory factors in LPS‐stimulated RAW264.7 cells, which exerted good anti‐inflammatory activity (Zhang et al. 2024). However, whether PLE and SA could also prevent the inflammatory diseases in vivo remains to be further explored. Thus, 5‐ to 6‐week‐old Balb/c mice were induced to have acute enteritis by intraperitoneal injection of LPS, and then treated with different doses of PLE and standard SA (Figure 3A). DAI score was employed to reflect the severity of IBD and to evaluate the inflammatory status of the LPS‐induced mice (Han et al. 2019). After injection with LPS for 3 h, the overall status, DAI of the mice was significantly increased along with weight loss, and all of the mice displayed depression, diarrhea, loose stools, and mostly fecal occult blood, which indicated the enteritis model was successful. The DAI of model group was decreased after 24 h, which may be due to the mouse's self‐defense mechanism in action, and PLE supplementation had little effect on the DAI index. Interestingly, the same content of SA as that in PLE had much more significant effect, and the DAI index of SA group was restored to a level equivalent to the control group, which indicated that SA might have a good protective effect on LPS‐induced enteritis (Figure 3B,C).

**FIGURE 4:**
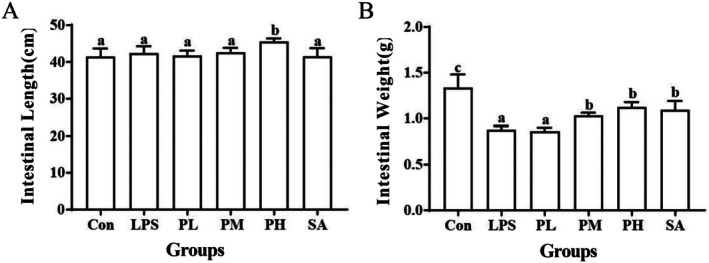
Effects of PLE and SA on small intestine length (A) and weight (B) of LPS‐induced mice.

It is commonly accepted that the length and weight of the small intestine are morphological indexes related to inflammation (Turgeon et al. 2013). The results indicated that although there was no significant difference in the intestinal length among groups (Figure 4A), the weight of the small intestine in the LPS group was significantly lower compared to the Con group (*p* < 0.05), which indicated that LPS caused a rise in inflammatory cell infiltration and disruption of the intestinal barrier function, leading to a reduction in small intestine weight (Yamazaki et al. 2004). In comparison to the LPS group, PLE and SA could significantly increase the small intestine weight, and this effect was attributed to its capability to effectively inhibit the intestinal stress response stimulated by LPS (Figure 4B).

**FIGURE 5:**
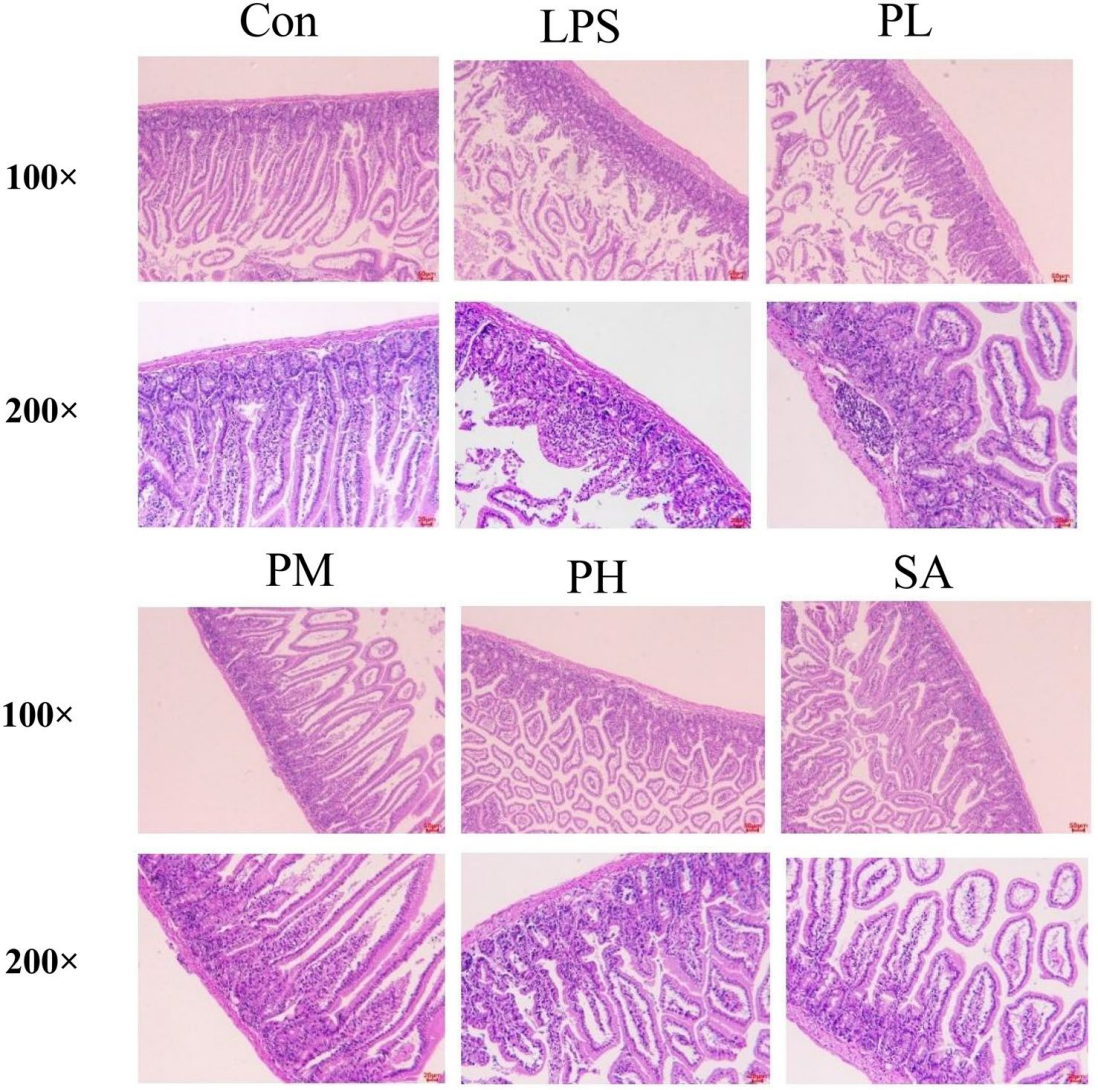
Representative photographs of H&E staining of the small intestine tissues.

In order to further research the therapeutic effects of PLE on LPS‐induced enteritis, pathological examinations of small intestine sections were performed using H&E staining. As shown in Figure 5, tissue sections from control group mice showed a histologically normal structure (Con), whereas intestinal mucosa induced by LPS showed severe histological changes, including villi shedding and inflammatory cell accumulation (LPS). However, this phenomenon was improved with an increase in PLE (PL, PM, and PH). In the PL group, there was still a small amount of inflammatory cell infiltration, and villus height was shortened, while the PM group improved the phenomenon significantly, similar to SA treatment (Chen et al. 2022; Ma et al. 2021). Furthermore, the epithelial cells appeared healthier and more intact, with fewer signs of necrosis or ulceration. All the morphological alterations indicated that PLE supplementation and SA could alleviate LPS‐induced enteritis symptoms.

### 3.3 Anti‐Inflammatory Mechanism of PLE on LPS‐Induced Enteritis

**FIGURE 6:**
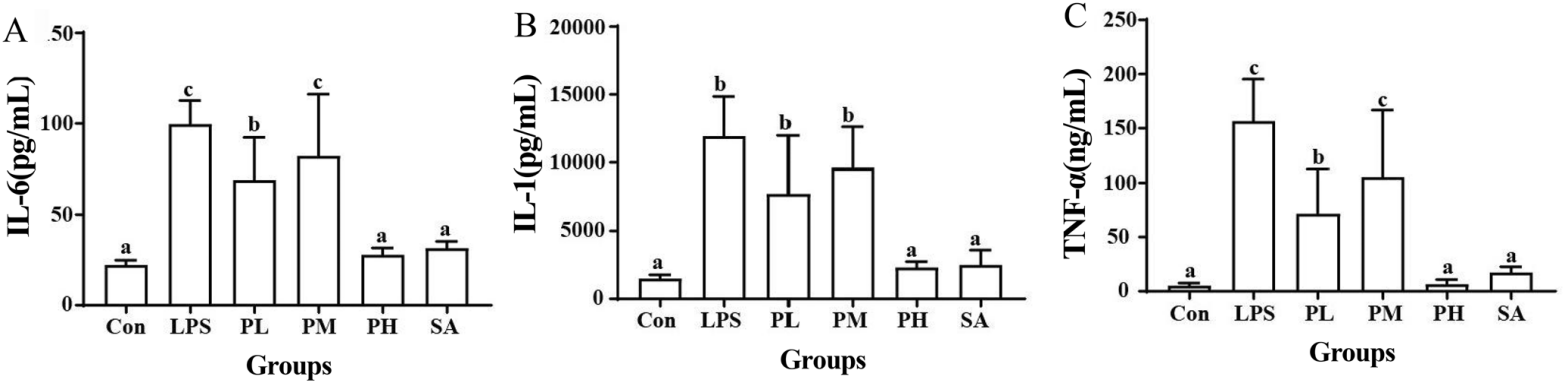
Effects of PLE and SA on the expression of TNF‐α (A), IL‐6 (B), and IL‐1β (C) in small intestine tissues.

Cytokines have a fundamental role in controlling mucosal inflammation in IBD. Lamina propria dendritic cells and macrophages are key antigen‐presenting cells that are found in the inflamed mucosa in IBD. Following activation, which occurs in response to components of the commensal microbiota and Toll‐like receptor (TLR) signaling, these cells produce large amounts of pro‐inflammatory cytokines, such as IL‐1β, IL‐6, and TNF‐α (Neurath 2014). To further clarify the mechanism underlying the therapeutic effect of PLE on IBD, the inflammation cytokine levels in the small intestine were determined. As shown in Figure 6, after intraperitoneal injection of LPS for 24 h, the levels of the pro‐inflammatory cytokines IL‐1β, IL‐6, and TNF‐α were significantly increased in the small intestine compared with those in the Con group. The addition of PLE effectively dose‐dependently reduced the production of TNF‐α, IL‐6, and IL‐1β, which was more significant in the high‐dose PLE (PH) group. In addition, the reduced levels of TNF‐α, IL‐6 and IL‐1β in PH group with 600 mg/kg extract were similar to those in the same content of the SA group, and both of them reached the normal level before modeling, which indicated that PLE could effectively alleviate LPS‐induced acute enteritis, and the main anti‐inflammatory active component was SA.

**FIGURE 7:**
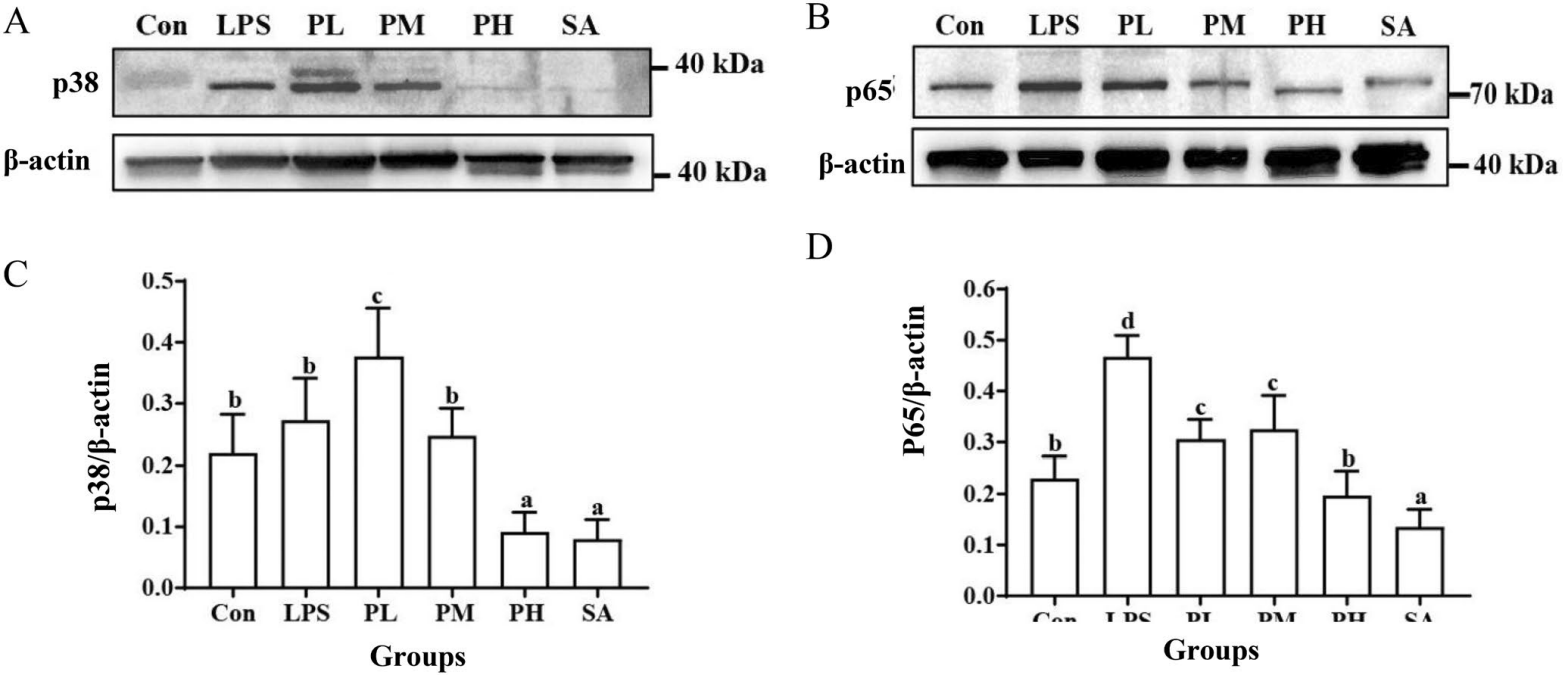
Effects of PLE and SA on the expression of p38 (A and C) and p65 (B and D) in small intestine tissues.

Toll‐like receptor 4 (TLR4), a mammalian receptor for bacterial LPS, plays a beneficial role in immune responses to bacterial infections but is also a main driver of aberrant inflammation. TLR4 can be activated by LPS and consequently promotes downstream signaling cascades to induce the secretion of inflammatory cytokines, including the activation of nuclear factor‐kappa B (NF‐κB) and mitogen‐activated protein kinases (MAPKs) family (Silva et al. 2016). Therefore, in order to further explore the anti‐inflammatory mechanism by which PLE inhibited the secretion of inflammatory cytokines, the expression levels of key signaling molecules in the NF‐κB and MAPK signaling pathways were assayed. Among them, the p38 MAPK signaling pathway plays an essential role in macrophage‐mediated inflammation, which can be activated by LPS, resulting in the stimulation of the production of TNF‐α, IL‐1β, and IL‐6 (Yang et al. 2014). As shown in Figure 7A,C, LPS slightly increased the expression of p38, while a high dose of PLE and its active component SA both inhibited the expression of p38 significantly. NF‐κB p65 played a predominant role in chronic intestinal inflammation, and NF‐κB nuclear translocation was often recognized as a cell reaction to LPS stimulation and was correlated with NF‐κB‐mediated transcriptional activation (Neurath and Pettersson 1997; Song et al. 2010). Therefore, we also determined whether PLE and SA could downregulate the expression of p65. As shown in Figure 7B,D, LPS stimulation led to an increase in p65 levels, while the treatment of different doses of PLE and SA all significantly downregulated the expression of p65. The results suggested that PLE could regulate the alteration in inflammatory cytokine production, which was associated with the inhibition of p38 MAPK and NF‐κB signaling pathways.

## 4 Conclusion

In this study, it was clarified that stearic acid was the main anti‐inflammatory substance in purslane. It could regulate the inflammatory response of LPS‐stimulated RAW264.7 macrophages mainly through the attenuation of inflammatory mediators NO, as well as the suppression of inflammatory factors TNF‐α, IL‐6, and IL‐10. In addition, it was demonstrated that PSA could attenuate LPS‐induced intestinal impairment by promoting the weight of the small intestine, alleviating aggravated small intestine histopathology, and inhibiting the expression of inflammatory factors. PLE could significantly reduce the levels of TNF‐α, IL‐6, and interleukin‐1β (IL‐1β) in the small intestine through the NF‐κB and MAPK signaling pathways. Summarily, our results highlight the beneficial effects of using purslane extracts to combat the LPS‐induced inflammatory response. Owing to its satisfactory biological activity, PSA is being considered as a promising candidate for the treatment of IBD, either as a dietary supplement or as a drug. Future studies using chronic or genetically modified IBD models would provide deeper insights into the precise molecular targets of stearic acid within the NF‐κB and MAPK pathways, thereby justifying detailed mechanistic elucidation, which lays a foundation for the anti‐inflammatory application of purslane extract in clinical.

## Author Contributions

**Xiao‐Min Lin:** data curation (equal), methodology (equal), writing – original draft (equal). **Ai‐Jing Chen:** data curation (equal), methodology (equal). **Hui‐Min Zhang:** formal analysis (equal), investigation (equal), visualization (equal). **Zhi‐Zhuo Kuang:** data curation (equal). **Rui Chen:** methodology (equal). **Meng‐Die Wu:** software (equal). **Jie Chen:** visualization (equal). **Xin Ma:** data curation (equal). **Lei Zhao:** conceptualization (equal), supervision (equal). **Yan Xing:** funding acquisition (equal), resources (equal). **He Ni:** project administration (equal), supervision (equal), writing – review and editing (equal).

## Conflicts of Interest

The authors declare no conflicts of interest.

## Data Availability

Data will be made available on request.
